# Usefulness of the New Hematological Parameter: Reactive Lymphocytes RE-LYMP with Flow Cytometry Markers of Inflammation in COVID-19

**DOI:** 10.3390/cells10010082

**Published:** 2021-01-06

**Authors:** Elżbieta Rutkowska, Iwona Kwiecień, Katarzyna Kulik, Beata Chełstowska, Krzysztof Kłos, Piotr Rzepecki, Andrzej Chciałowski

**Affiliations:** 1 Laboratory of Hematology and Flow Cytometry, Department of Internal Medicine and Hematology, Military Institute of Medicine, 04-141 Warsaw, Poland; ikwiecien@wim.mil.pl (I.K.); kkulik@wim.mil.pl (K.K.); 2 Faculty of Medicine, Collegium Medicum, Cardinal Stefan Wyszynski University, 01-815 Warsaw, Poland; beata-chelstowska@wp.pl; 3 Department of Infectious Diseases and Allergy, Military Institute of Medicine, 04-141 Warsaw, Poland; kklos@wim.mil.pl (K.K.); achcialowski@wim.mil.pl (A.C.); 4 Department of Internal Medicine and Hematology, Military Institute of Medicine, 04-141 Warsaw, Poland; przepecki@wim.mil.pl

**Keywords:** reactive lymphocytes, COVID-19, flow cytometry, CD25, CD45RO, HLA-DR, CD38, plasmablasts

## Abstract

Identification of patients with activation of the immune system which indicates the presence of infection is essential, especially in the times of the global coronavirus 2019 (COVID-19) pandemic. The aim of the present study was to evaluate the reactive lymphocytes (RE-LYMP) parameter in COVID-19 and to correlate it with activation lymphocytes markers by flow cytometry. The study group consisted of 40 patients: with COVID-19 infection (*n* = 20) and with others virus infections without COVID-19 (COVID-19(−) virus (*n* = 20)) and 20 healthy donors (HC). Blood count and flow cytometry were performed. The COVID-19(+) group had significantly lower RE-LYMP parameter than the COVID-19(−) virus group (5.45 vs. 11.05, *p* < 0.05). We observed higher proportion of plasmablasts in the COVID-19(+) and COVID-19(−) virus groups than HC (8.8 vs. 11.1 vs. 2.7, *p* < 0.05). In the COVID-19(+) there was a lower proportion of CD4+ CD38+ cells than in the other groups (significant differences between COVID-19(+) and COVID-19(−) virus groups). RE-LYMP correlated with activated T lymphocytes CD38+ and HLA-DR+ in the COVID-19(−) virus group, however in the COVID-19(+) group correlations with T lymphocytes CD25+ and CD45RO+ were observed. In summary the analysis of the RE-LYMP together with flow cytometric activation markers can be helpful in identifying and distinguishing patients with COVID-19(+) from other viruses and HC.

## 1. Introduction

Inflammatory diseases associated with infection of various types of viruses are nowadays very common. The rate of mutation of individual types of pathogens and the fact that a large number of infections are asymptomatic or atypical, pose a huge challenge to the medical staff involved in the diagnosis of this type of disease. This is especially important in the era of the new coronavirus disease 2019 (COVID-19) global pandemic [1]. Immune response is heterogeneous dependent of type of virus, severity of the disease and remains poorly understood. Clinicians need to have fast, cheap and routine parameters to indicate dysregulation of the immune system.

As a result of recent technological advances, today’s hematological analyzers allow the determination of not only routine complete blood count but also new additional parameters, which could help in evaluation of selected inflammatory diseases. Use of these new hematological parameters requires additional further research.

The Sysmex XN-Series hematological analyzers (Sysmex Corp., Kobe, Japan) allow differentiation of activated lymphocytes among all leukocytes as a new parameter called reactive lymphocytes (RE-LYMP). This process is carried out by fluorescence emission based on the combination of side scatter (inner complexity of the cell), forward scatter (size of the cell), and fluorescence intensity (RNA content) of nucleated cells [2,3]. Activated lymphocytes have higher fluorescence intensity than normal lymphocytes [4]. The presence and percentage of RE-LYMP may be a very crucial in indicating the inflammation process in the patient’s blood as well as providing clinicians with additional information about activation of the immune response. The RE-LYMP parameter reflects all lymphocytes that have a higher fluorescence signal than the normal lymphocyte population. The change of value in the RE-LYMP parameter depends on the severity and stage of the infection [5]. However, accurate determination of cell subpopulations, including lymphocytes in the blood of the patients is allowed only with the flow cytometry method. We hypothesized that additional and rapid lymphocyte parameters such as RE-LYMP could correlate with “activation lymphocyte antigens” obtained by flow cytometry and may provide useful information of the status of the immune system during infection, especially in COVID-19 patients.

Activated lymphocytes are known to express on their surface a number of molecules termed ‘‘activation antigens’’ which could be easily detected by multiparameter flow cytometric analysis using monoclonal antibodies as specific reagents. For our study, we chose T cell activation molecules which are expressed during very early CD25 and mid-to-late antigens: CD45RO, CD38 and HLA-DR time periods after stimulation on both CD4+ and CD8+ lymphocytes. For B cell activation analysis we examined cells named plasmablasts with high expression of CD38 markers. Expression of these markers is under complex control and varies during lymphocyte development, activation and differentiation, suggesting an important role of these processes [6,7].

CD25 is the alpha chain of the trimeric IL-2 receptor and considered to be the most prominent cellular activation marker. It is expressed constitutively on the surface of several subsets of peripheral blood lymphocytes, such as regulatory and resting memory T cells. It is upregulated within 24 h of stimulation of the TCR/CD3 complex and remains elevated for a few days [8].

In peripheral blood, the CD45RO antigen is present on approximately 40% of resting peripheral blood T lymphocytes, including the CD4+ and CD8+ subpopulations, as well as on most thymocytes and activated T lymphocytes [9,10]. Upon activation naive T lymphocytes first acquire CD45RO and then lose CD45RA [11]. When these activated T lymphocytes are rechallenged, the cells that exhibit a secondary response are primarily CD45RO+, leading to the concept that CD45RO+ cells are a primed population of memory T lymphocytes.

CD38 is a multi-functional transmembrane protein present on the lymphoid cells and highly expressed on early T and B cell precursors [12,13,14]. In contrast, mature lymphocyte cells have low levels of CD38, but upon activation, they up-regulate its expression both on T and B lymphocytes. Activated B cells proliferate and differentiate into antibody-producing plasmablasts and long-lived plasma cells expressing CD38 at high level [15].

HLA-DR is a human class II major histocompatibility complex (MHC) antigen which is constitutively expressed on the surface of B lymphocytes, monocytes, and macrophages and appears at the late stages of activation on T and NK cells; thus, it is considered to be a very late activation marker [16].

Considering that the RE-LYMP parameter is quick and easy to determine, and may reflect the lymphocyte antigen expression, which was discussed above, we decided to evaluate the RE-LYMP parameter in the inflammatory response in COVID-19 patients and other infectious diseases and to correlate it with activation lymphocyte markers assessed by the flow cytometry method.

## 2. Material and Methods

### 2.1. Study Design and Participants

The study group consisted of 40 patients undergoing a routine peripheral blood (PB) test with a virus infectious disease and 20 healthy controls (HC), from 10 May to 30 June 2020 at the Military Institute of Medicine (Department of Internal Medicine and Hematology, Laboratory of Hematology and Flow Cytometry and the Department of Infectious Diseases and Allergology).

The study group consisted of: patients with a COVID-19 positive test (*n* = 20, COVID-19(+)) confirmed by real-time reverse transcriptase–polymerase chain reaction (RT-PCR) assay for nasal and pharyngeal swab specimens according to the WHO guidelines and patients with virus infections without COVID-19 (*n* = 20, COVID-19(−) viruses).

PB samples were collected from all patients. Demographic details of patients with virus infections are presented in Table 1. Table 1 shows the total number of patients with viruses, age, gender, clinical symptoms, characterization of viruses, and routine blood count with differential and additional inflammatory Sysmex parameters.

Patients with an unequivocally positive COVID-19 test were newly admitted (Department of Infectious Diseases and Allergology, Military Institute of Medicine), mainly without treatment and respiratory distress, with stable lung parameters and oxygen supplementation in a few cases and without signs of pulmonary congestion or fluid in the pleural cavities (COVID-19 patients’ characteristics are presented in Appendix A: Table A1).

The blood samples used in the study were taken during routine diagnostics and were approved by the Ethics Committee of the Military Institute of Medicine who gave informed consent in light of the urgent need to collect clinical data (Military Institute of Medicine Ethics Committee number: 47/WIM/2020).

### 2.2. Data Collection

Data including demographic information, medical history, symptoms, signs, and laboratory findings were collected from patients’ medical records. Laboratory results included blood routine tests were made with the use of the Sysmex XN-1500, lymphocyte subsets and infection-related biomarkers.

In addition to the routine blood count parameters, such as: white blood cell count (WBC), neutrophils, lymphocytes, monocytes, eosinophils, basophils, platelets (PLT) counts and immature granulocytes (IG), the study evaluated the research parameter: RE-LYMP using the Sysmex XN-1500.

Leukocyte subset percentage and infection-related biomarkers were performed by the flow cytometry method with a panel of monoclonal antibodies using FACS Canto II BD flow cytometry (Becton Dickinson, Franklin Lakes, NJ, USA). For surface markers detection on the leukocyte subset and infection-related biomarkers, cells were stained with fluorescently labelled antibodies: CD4-FITC (catalog number: 345768, clone number: SK3), CD38-PE (catalog number: 555460, clone number: HIT2), CD3-PerCP-Cy5.5 (catalog number: 332771, clone number: SK7), CD8-APC (catalog number: 345775, clone number: SK1), CD16-APC-H7 (catalog number: 560195, clone number: 3G8), CD19-FITC (catalog number: 363007, clone number: SJ25C1), CD25-APC (catalog number: 340907, clone number: 2A3), CD45RO-PE-Cy7 (catalog number: 560608, clone number: UCHL1), CD38-APC-H7 (catalog number: 656646, clone number: HB7), HLA-DR-V450 (catalog number: 655874, clone number: L243), CD45-V500 (catalog number: 655873, clone number: 2D1), CD19-PE-Cy7 (catalog number: 341113, clone number: SJ25C1) (BD Biosciences) for 20 min at room temperature. After two washings, cells were analyzed within 2 h. For each sample, a minimum of 20 000 events were collected. Data were analyzed with DIVA Analysis software 8.0.1 (BD) and Infinicyt 1.8 Flow Cytometry (Cytognos, Salamanca, Spain).

The data were reviewed by a trained team of medical scientists and physicians in the Military Institute of Medicine (Laboratory of Hematology and Flow Cytometry, Department of Internal Medicine and Hematology and Department of Infectious Diseases and Allergology).

### 2.3. Statistical Analysis

All statistical analyses were performed using the Statistica 13.0 software (TIBCO Software, Palo Alto, CA, USA). *p* values of less than 0.05 were considered statistically significant. The results were expressed as medians (Q1–Q3) and the median of geometric means of fluorescence (GMF) intensity of reactive markers on lymphocytes. For group comparison the Kruskal–Wallis ANOVA test and post-hoc analysis test were used. Relations between the quantitative variables were analyzed by Spearman correlations.

## 3. Results

### 3.1. Characteristics of Study Groups: Clinical and Blood Count Parameters

**Figure A1:**
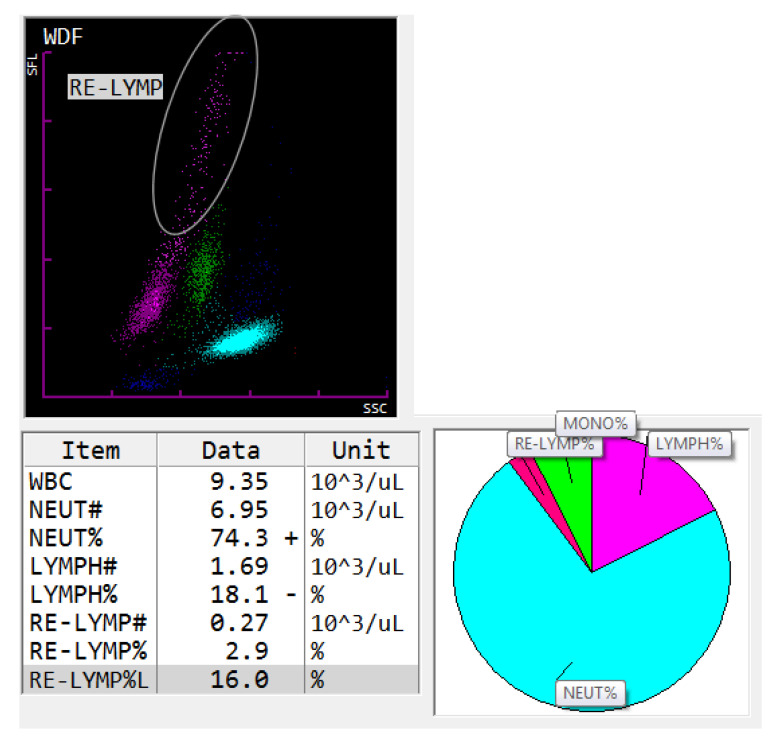
Sample screenshot from the Sysmex XN-1500 analysis software showing the distribution of leukocytes (plot SSC vs. SFL) with selected reactive lymphocytes (RE-LYMP).

The clinical characteristics of the investigated group with virus infections including the routine blood count are summarized in Table 1. The routine blood count, such as: WBC, neutrophils, lymphocytes, monocytes, eosinophils, basophils, PLT counts, and IG were normal in the group with virus infection (together COVID-19 and other virus infection) compared to the Sysmex reference values [17]. Additionally, the study evaluated RE-LYMP using the Sysmex research parameters (Sample screenshot from the Sysmex XN-1500 analysis software showing selected RE-LYMP is presented in Appendix A: Figure A1). The RE-LYMP median proportion was slightly higher in the study group with viruses than in the reference group (Sysmex specification standard 0–5% RE-LYMP [3]). In addition, the minimum and maximum range of this parameter was 1.2–52.9%.

Patients were divided into two groups: with COVID-19 positive test (*n* = 20) and without COVID-19 infection with other virus infections (*n* = 20) (Table 1 presented the characteristics of viruses) and compared to HC (*n* = 20). It was observed that patients with COVID-19(+) had a lower median of absolute count of lymphocytes, eosinophils, and basophils than HC and only the lower count of lymphocytes differed from the other virus infection group (COVID-19(−) virus group) (Table 2). Absolute counts of median proportion of Sysmex morphological parameters such as: WBC, neutrophils, lymphocytes, monocytes, eosinophils, basophils, IG and PLT did not differ between COVID-19(−) viruses and HC. COVID-19(+) had a significantly lower RE-LYMP parameter than the COVID-19(−) virus group (5.45 vs. 11.05, *p* < 0.05) and a slightly but non-significant elevation compared to the Sysmex reference values (Sysmex specification standard 0–5% RE-LYMP [3]) and to the HC groups (5.45 vs. 4.20, *p* > 0.05).

### 3.2. Analysis of Leukocyte and Plasmablast Subpopulations by Flow Cytometry

In the next step, all three groups were compared using the assessment of leukocyte subpopulations by flow cytometry (Table 3). When we analyzed the proportion of plasmablast we observed a higher proportion in COVID-19(+) patients and the COVID-19(−) virus group than the HC group (respectively, 8.8 vs. 11.1 vs. 2.7, *p* < 0.05). We did not observe differences in the plasmablast median proportion between COVID-19(+) patients and the COVID-19(−) virus group. The lowest median proportion of the absolute count of lymphocytes was observed in COVID-19(+) patients compered to the HC group and the COVID-19(−) virus group (respectively, 0.98 vs. 2.16 vs. 2.25 × 10^3^/µL, *p* < 0.05). However, we did not observe any differences in basic leukocyte subpopulation between the three groups using the flow cytometry method (Table 3).

### 3.3. Lymphocyte T Subtypes with Expression of Activation Markers: CD25, CD45RO, CD38 and HLA-DR

**Figure 1:**
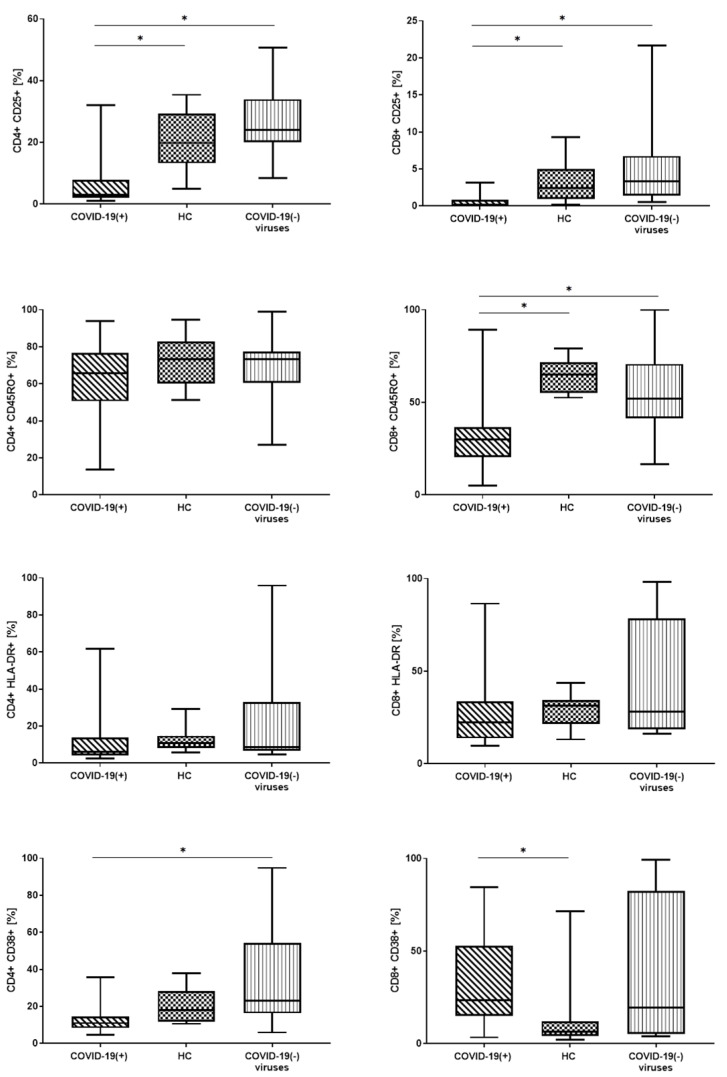
The differences of lymphocyte T subtypes with expression of activation markers CD25, CD45RO, CD38 and HLA-DR between patients: with positive COVID-19 test (COVID-19(+)), control group (HC), and group with other virus infections with negative COVID-19 test (COVID-19(−) virus). Graphs show the median values (Min–Max), * *p* < 0.05.

**Figure A2:**
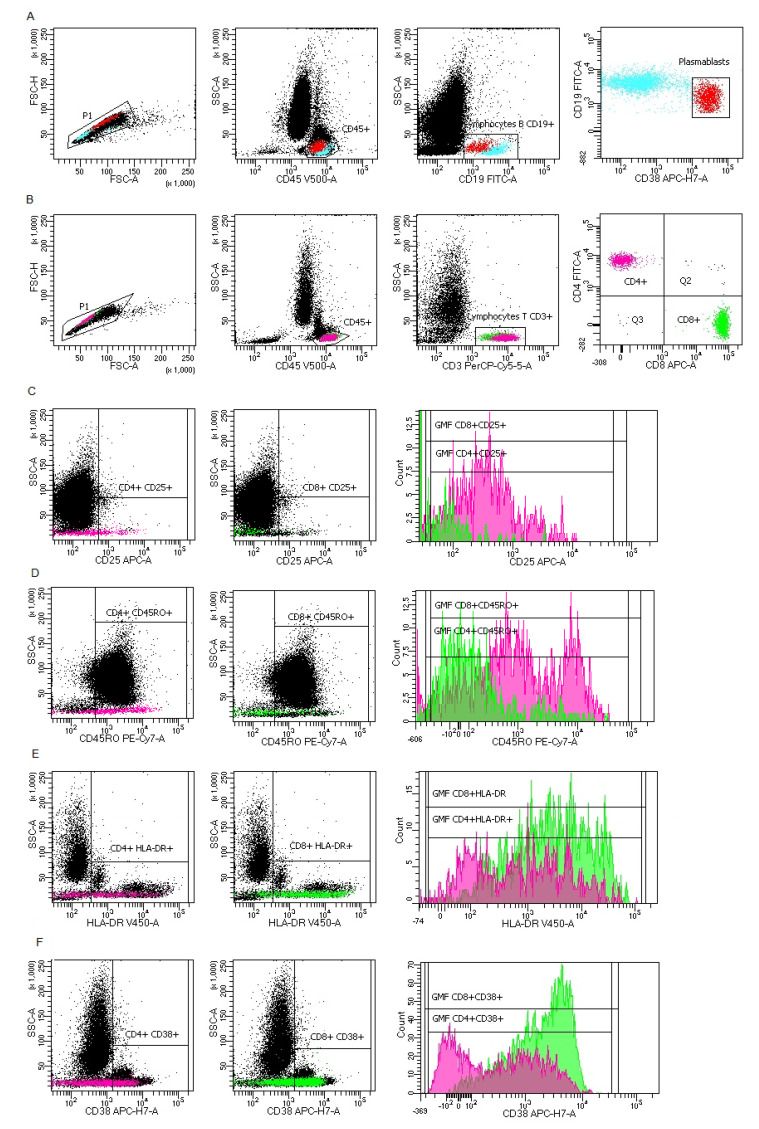
Representative FACS analysis of PB cells with antibodies specific for plasmablasts and T cells (CD4+ and CD8+) with CD25, CD45RO, CD38 or HLA-DR expression. (**A**) Plasmablast gating strategy: FSC-A vs. FSC-H plot: Gating the cells that have an equal area and height, thus removing clumps (greater FSC-A relative to FSC-H) and debris (very low FSC), CD45 vs. SSC-A plot: Selection of lymphocytes based on their SSC/CD45+ properties, CD19 vs. SSC-A plot: Selection of lymphocytes B (blue) based on their SSC/CD19+ properties, CD19 vs. CD38 plot: Selection of plasmablasts (red) based on their CD19/CD38 properties (CD19+ and CD38+high). (**B**) T lymphocyte gating strategy: FSC-A vs. FSC-H plot: Gating the cells that have an equal area and height, thus removing clumps (greater FSC-A relative to FSC-H) and debris (very low FSC), CD45 vs. SSC-A plot: Selection of lymphocytes based on their SSC/CD45+ properties, CD3 vs. SSC-A plot: Selection of lymphocytes T based on their SSC/CD3+ properties, CD8 vs. CD4 plot: Selection of T lymphocyte subpopulation: CD4+ (pink) and CD8+ (green) based on their CD4/CD8 properties. (**C**) Representative plots with CD25 proportion and CD25 geometric mean fluorescence (GMF) intensity on CD4+ and CD8+ T lymphocyte subpopulation. (**D**) Representative plots with CD45RO proportion and CD45RO GMF intensity on CD4+ and CD8+ T lymphocyte subpopulation. (**E**) Representative plots with HLA-DR proportion and HLA-DR GMF intensity on CD4+ and CD8+ T lymphocyte subpopulation. (**F**) Representative plots with CD38 proportion and CD38 GMF intensity on CD4+ and CD8+ T lymphocyte subpopulation.

Next, in these three groups: COVID-19(+), HC, and COVID-19(−) virus group we analyzed median proportion and GMF intensity of: CD25, CD45RO, CD38, and HLA-DR markers on CD4 and CD8 lymphocytes T. We compared the differences in expression of these antigens between these three groups (Figure 1, Table 4). Representative FACS analysis of PB cells with antibodies specific for plasmablasts and T cells (CD4+ and CD8+) with CD25, CD45RO, CD38 or HLA-DR expression are presented in Appendix A, Figure A2.

In the COVID-19(+) patients we noticed the lowest median proportion of CD25+ CD4+, and CD8+ CD25+ cells compared to the HC group and COVID-19(−) virus group (respectively for CD4+ CD25+: 3.0 vs. 19.8 vs. 24.1, for CD8+ CD25+: 0.2 vs. 2.5 vs. 3.4, *p* < 0.05) (Figure 1, Table 4). When we analyzed the GMF intensity of CD25+ on CD4+ and CD8+ cells we also observed the lowest proportion in patients with COVID-19(+) (Table 4).

There were not any differences in the median proportion of CD45RO+ CD4+ cells between the three groups (Figure 1), but when we analyzed the median of CD4+ CD45RO+ GMF intensity we observed the lowest proportion in COVID-19(+) patients (Table 4). We observed the lower median proportion of CD8+ CD45RO+ cells and lower median of CD8+ CD45RO+ GMF intensity in COVID-19(+) patients compared to the HC group and COVID-19(−) virus group (Table 4).

We did not observe any differences between the three groups in the median proportion of HLA-DR+ and GMF of HLA-DR on CD4+ and CD8+ (Table 4, Figure 1).

In the COVID-19(+) we noticed a lower median proportion of CD4+ CD38+ cells than in the HC group and COVID-19(−) virus group (significant differences between COVID-19(+) and COVID-19(−) virus group, (respectively 11.0% vs. 23.0%, *p* < 0.05) (Figure 1, Table 4). We did not observe differences between the three groups when we analyzed the GMF of CD38 on CD4+ (Table 4).

The median proportion of CD8+ CD38+ cells and CD8+ CD38+ GMF intensity analysis showed the highest proportion in COVID-19(+) patients and significant differences between COVID-19(+) patients and the HC group (Figure 1, Table 4).

### 3.4. Correlations between the RE-LYMP Parameter and Activation Lymphocyte Markers

**Figure 2:**
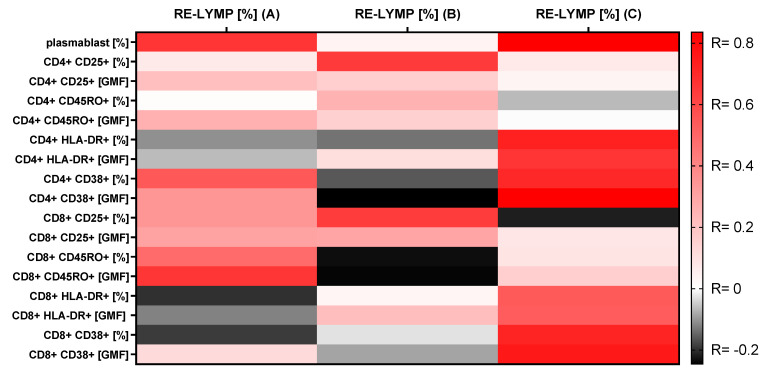
A heatmap of Spearman correlation coefficients for the RE-LYMP parameter with activation lymphocytes markers using multiparameter flow cytometry in the 3 groups. A. with positive COVID-19 test, B. control group (HC), C. group with other virus infections with negative COVID-19 test. Correlations with an absolute value more than 0.5 are associated with *p* < 0.05, red—positive correlations, black—negative correlations.

We observed a positive significantly strong correlation between RE-LYMP parameter and plasmablast proportion in COVID-19(+) patients (R = 0.7, *p* < 0.05) and the COVID-19(−) virus group (R = 0.8 *p* < 0.05) and without this correlation in the HC group (Figure 2).

In the COVID-19(+) group there was a significantly positive correlation between the percentage of RE-LYMP and CD4+ CD38+ lymphocytes (R = 0.5, *p* < 0.05) and between the percentage of RE-LYMP and GMF CD8+ CD45RO intensity (R = 0.7, *p* < 0.05).

In the COVID-19(−) virus group there was a high positive correlation between the percentage of RE-LYMP and proportion of CD4+ CD38+ cells (R = 0.7, *p* < 0.05) and between the percentage of RE-LYMP and GMF CD4+ CD38+ intensity (R = 0.8, *p* < 0.05). Moreover, in this group we also observed a high positive correlation between the percentage of RE-LYMP and proportion of CD4+ HLA-DR+ cells (R = 0.7, *p* < 0.05) and between the percentage of RE-LYMP and GMF CD4+ HLA-DR+ intensity (R = 0.6, *p* < 0.05). Significantly positive correlations between the percentage of RE-LYMP and proportion of CD8+ CD38+ cells (R = 0.7, *p* < 0.05) and between the percentage of RE-LYMP and GMF CD8+ CD38+ intensity (R = 0.7, *p* < 0.05) were observed. Moreover, in this group there was observed a high positive correlation between the percentage of RE-LYMP and the proportion of CD8+ HLA-DR+ cells (R = 0.5, *p* < 0.05) and between the percentage of RE-LYMP and GMF CD8+ HLA-DR+ intensity (R = 0.5, *p* < 0.05) (Figure 2).

In the HC group we observed a significant positive correlation between RE-LYMP and the proportion of CD4+ CD25+ cells and between RE-LYMP and the proportion of CD8+ CD25+ cells. (Figure 2). All correlations between the RE-LYMP parameter with activation lymphocyte markers in the three groups are presented in Figure 2.

## 4. Discussion

The evaluation of the RE-LYMP parameter in the inflammatory response in COVID-19 and other infectious diseases and correlations with activation markers in flow cytometry was discussed in the present work. In our study, we focused on the assessment of the RE-LYMP parameter as a screening parameter in COVID-19 patients.

### 4.1. Basic Morphological Parameters with Reactive Lymphocytes (RE-LYMP)

Patients were divided into two groups: COVID-19 positive test (COVID-19(+)) group and with virus infections group without COVID-19 infection (COVID-19(−) viruses) and compared to the HC group. The study group consisting of patients with viral infections (COVID-19(+) and other viruses) is characterized in Table 1.

We observed that patients with COVID-19(+) infection had the lowest number of lymphocytes, eosinophils, and basophils. These results are consistent with those presented by other researchers, which confirmed lymphopenia in COVID-19 patients [18,19,20,21,22]. The median proportion of RE-LYMP in COVID-19(+) patients was significantly lower than the COVID-19(−) virus group (5.45 vs. 11.05, *p* < 0.05) and slightly elevated compared to the HC group. It has shown that the RE-LYMP parameter together with basic morphological parameters such as lymphopenia, basopenia, and eosinopenia allowed patients with COVID-19(+) infection in a moderate state to be distinguished from the other virus infection and HC groups. However, the RE-LYMP parameter in the COVID-19(+) group may be heterogeneous and its level could depend on the severity of the disease. Yip, C. et al. [5] showed that in patients with severe/critical COVID-19, RE-LYMP was higher than in mild cases. This suggests that the RE-LYMP parameter, not only distinguishes COVID-19(+) from other viral infections in moderate cases, but may also be a marker of disease severity. We did not observe other differences between the three groups with regard to the routine blood count parameters obtained from Sysmex.

It is known that lymphocytes and their subsets play an important role in maintenance of the immune system function. As with immune diseases and other infectious disease, virus infection can lead to dysregulation in the level of lymphocyte subsets [23,24]. In COVID-19(+) patients some researchers revealed activation and proliferation of lymphocytes T and plasmablast infiltration [25], but immune characteristic of this response could be heterogenous and remain poorly understand.

### 4.2. Basic Lymphocyte Subpopulations by Flow Cytometry

In this study we observed differences only in the absolute count of lymphocytes and without other differences in lymphocyte subpopulations between three groups: COVID-19(+), HC group, and COVID-19(−) virus using the flow cytometry method (Table 3). In another study Wang F. et al. [26] reported that patients with a severe case had significantly lower total lymphocytes, CD4+, CD8+ cells and B cells, however similar to our study no significant difference was observed in the CD4/CD8 ratio and NK cells. Other researchers also noted that differences in the percentage of basic leukocyte subpopulations may be related to the severity of the disease and manifest themselves only in severe forms of COVID-19 infection. Moratto D. et al. [27] showed that flow cytometry analysis revealed significant differences among patients with moderate disease, those with a severe phenotype who eventually recovered, and those who progressed to a critical phenotype. Liu, Z. et al. [28] in their interesting study also found that low counts of CD4+ and CD8+ lymphocytes T were more common in patients with severe COVID-19 and the CD4/CD8 ratio showed no significant difference between the non-severe and severe COVID-19 groups. In our study, we observed only a significant decrease of the absolute count of lymphocyte population in COVID-19(+) patients compared to other infectious diseases and HC. This finding could be attributed to the fact that most of the patients in our study were of moderate infection.

Therefore, it seemed reasonable to look for other markers that could be used in the diagnosis of patients with inflammation, especially with mild and non-severe COVID-19 infection. Others have revealed activation of T cell and B cell subsets in a proportion of patients with COVID-19. A subgroup of patients had T cell activation characteristic of acute viral infection and plasmablast responses reaching >30% of circulating B cells [25]. However, another subgroup had lymphocyte activation comparable to uninfected subjects.

### 4.3. Plasmablast and Activation T Lymphocyte Markers

We noted that patients with other viruses without COVID-19(−) infection and patients with COVID-19(+) had a significantly higher proportion of plasmablasts than HC, but these two groups did not differ statistically from each other. Due to the presence of differences between the whole study group and HC in the percentage of plasmablasts, these cells may be used as an identification marker of immune system activation. In our study, we showed for the first time the correlation between plasmablasts and the new hematological parameter RE-LYMP. Our observations showed that the RE-LYMP parameter can reflect the level of plasmablasts assessed by flow cytometry and that the differences in the percentage of plasmablasts could depend on the number of reactive lymphocytes. Kuri-Cervantes, L. et al. [29] observed that the activated lymphocytes in COVID-19 infection correlated with the proportion of the plasmablasts, but the activated lymphocytes were measured by the flow cytometry method, not by the hematological parameter RE-LYMP. Other researchers have shown that in natural viral infections, very impressive numbers of plasmablasts in the blood are generated [30,31,32]. However, in our study, the lack of differences in the number of plasmablasts between patients with other infections and COVID-19 did not make it possible to distinguish between these groups.

In our study, we decided to analyze by the flow cytometry method the common and known markers of inflammation and indicators of cell T activation CD25, CD45RO, CD38, and HLA-DR [6,33,34,35]. A number of studies demonstrated that an adaptive immunity responding to coronavirus is required for efficient clearance of the virus. In patients infected with COVID-19, the acute phase of infection in humans was associated with reduction of lymphocyte number in the blood, involving a loss of T cells in comparison to healthy control individuals [36,37]. This suggests that COVID-19 infection impaires cellular immunity and indicates that T cell activation in response to the virus is impaired [6,38].

We observed a statistically lower percentage of CD4+ CD25+ and CD8+ CD25+ cells in the COVID-19(+) group compared to boththe HC and COVID-19(−) virus group. The CD25 molecule is a receptor for IL-2 that has a variety of functions promoting proliferation, differentiation of activated CD4+ and CD8+ T cells, while also inducing T regulatory lymphocyte (Tregs) cell survival [39]. We hypothesized that the decrease of CD25+ positive cells was due to lack of Tregs cells in patients with COVID-19(+) infection. Moreover, several CD8+ Treg subsets have recently been identified including CD8+ CD25+ with properties similar to the functions and phenotype of CD4+ CD25+ [40]. Chen, G. et al. [41] confirmed our observation by showing reduced frequencies of Tregs in moderate and severe COVID-19 cases. Conversely, others observed elevated Tregs in mild patients, however in severe patients, the Tregs cellularity was comparable to that in the control individuals [42]. The assessment of the role of Tregs in COVID-19 infection requires further research on numerous groups at different stages of COVID-19 infection with exact identification of these cells.

Our research has shown a reduction of CD45RO+ positive cell percentage among CD8+ T cells with no group difference among CD4+ cells. Most naïve human T cells express a form of CD45RA, whereas memory T cells express a different isoform called CD45RO. Memory T cells have the ability to survive for long periods of time and are responsible for the rapid responses on subsequent exposure to antigen CD45RO [43]. In our previous study we observed that in patients with COVID-19 infection, lymphocytes CD8+ manifested effector profile, meanwhile lymphocytes CD4+ directed to memory profile [44]. In our opinion, the lower expression of CD45RO molecules on CD8+ cells may indicate undeveloped or impaired cell memory in COVID-19 patients, as opposed to CD4+ cells, where the expression of this molecule was at the level of the control group.

During analysis of the CD38+ marker on CD4+ cells we observed that there were lower CD4+ CD38+ cells in COVID-19(+) patients compared to the HC group and COVID-19(−) virus group. Interestingly, patients with COVID-19(+) infection and patients with COVID-19(−) virus were statistically significantly different. It seemed, therefore, that low levels of CD4+ CD38+ cells in COVID-19(+) patients could distinguish these patients from other inflammatory diseases. Song C. B., et al. [45] have shown that CD38 promotes proliferation in the CD4+ T cells of HIV-infected individuals. High levels of human virus-specific CD38+ CD4+ T cells have been reported to be transiently present during acute presentations also of other infections such as: CMV or EBV infections [1,46]. However, most studies of the role of CD38 antigen on CD4+ cells in antiviral response have focused on HIV infection [47,48,49]. Our observation of the decline of CD38+ CD4+ cell population in COVID-19(+) patients relative to other infections was shown here for the first time. In our previous study we observed a significant lower median absolute count of CD4+ cells in patients with COVID-19(+) compared to healthy control while the CD4+ cell had mainly memory profile not effector profile [44].

Taking into account the proportion of CD8+ CD38+ cells in the three studied groups, we noticed the highest percentage and GMF intensity of CD38 in COVID-19(+) patients, while due to the lack of statistical differences between COVID-19(+) patients and the COVID-19(−) virus group, the CD8+ CD38+ cell analysis did not separate COVID-19(+) patients on this basis. Our above observation suggested that CD8+ CD38+ cell responses in COVID-19(+) patients were similar to other viral infections. Benito J., et al. [50] have shown that levels of CD38+ on CD8+ cells increased in chronic HIV infection, and were strongly correlated with plasma viremia and the slow decline of CD38 expression on CD8+ cells over time after antiretroviral therapy. Zidovec Lepej, S. et al. [51] also analyzed the molecules CD38 on bright CD8+ T lymphocytes in CMV and EBV infections. This study has shown that acute EBV and CMV infections increased the numbers of CD38 molecules expressed on CD8+ T lymphocytes of patients compared to healthy controls. These results suggest that the mechanism of action by CD8+ CD38+ cells in viral infections, including COVID-19(+), may be similar, but it does not allow the type of infection to be distinguished.

When we analyzed the median proportion of HLA-DR+ and GMF of HLA-DR on CD4+ and CD8+ cells unexpectedly we did not observe any differences between the three groups (Table 4). It is known that during viral infection significant numbers of T cells are activated and their effector function and their potential roles in immunity to infection and immunopathology are underlined [52]. We expected similar results for HLA-DR as for CD38 expression, as it is known that in most infections there is a simultaneous increase in the expression of these two markers s [53,54,55].

Next, we analyzed the correlation between the above activation markers on CD4+ and CD8+ cells with the new hematological rapid parameter- RE-LYMP. We found RE-LYMP to be an interesting parameter to be used in the quick diagnosis of patients with infection, especially with COVID-19. We hypothesized that correlating it with other activation markers that can be tested by flow cytometry would facilitate the diagnosis and separation of COVID-19(+) patients from HC and other virus diseases with reactive lymphocytes before obtaining the PCR test result. In the study, we showed that in the group of COVID-19(−) virus there were the strongest positive correlations RE-LYMP with CD38 and HLA-DR markers in both CD4 and CD8+ cells without correlations with markers CD25 and CD45RO. Interestingly in the COVID-19(+) group there were positive correlations RE-LYMP with CD25 and CD45RO markers in both CD4 and CD8+ cells without correlations with markers CD38 and HLA-DR. The difference can be illustrated by heatmaps (Figure 2), moreover both viral groups clearly differ from the HC.

The above results confirmed that the RE-LYMP parameter with basic morphological parameters can reflect the activation state in virus infections, however an additional analysis of activation markers by flow cytometry together with the RE-LYMP parameter will allow to patients with COVID-19 be distinguished from other viruses and HC.

## 5. Conclusions

In summary, in our study the usefulness of the RE-LYMP parameter in the rapid diagnosis of viral infections was investigated. We confirmed that the RE-LYMP parameter may be a valuable tool for observing infected patients and provides evidence that further studies on the role of lymphocyte subsets are necessary. However, in COVID-19 patients, only the combination of activation markers tested by flow cytometry can provide useful information.

## Figures and Tables

**Table 1:** Demographic and laboratory data of patients with virus infections.

	Patients *n* = 40	
Sex: f/m (*n*)	25/15	
Age (mean ± SD years)	46.2 ± 19.1	
Women (mean ± SD years)	46.1 ± 17.3	
Men (mean ± SD years)	46.4 ± 24.1	
Clinical symptoms and diseases comorbidities (%) (no/yes)		
- fever	35.7/64.3	
- non-productive cough	65.2/34.8	
- myalgia	72.1/27.9	
- diabetes	84.3/15.7	
- hypertension	88.6/11.4	
- respiratory failure	97.2/2.8	
Groups (*n*)		
- COVID-19(+)	20	
- COVID-19(−) viruses:	20	
Epstein-Barr virus (EBV)	*9*	
Cytomegalovirus (CMV)	*6*	
Human immunodeficiency virus (HIV)	*2*	
Toxoplasmosis	*1*	
Influenza virus	*2*	
**Sysmex parameters** [10^3^/µL] [median (Q1–Q3)]		Reference values [3,17]
- WBC [10^3^/µL]	5.96 (3.90–7.89)	3.9–9.5
- NEUTROPHILS [10^3^/µL]	3.24 (2.39–4.34)	1.53–4.98
- LYMPHOCYTES [10^3^/µL]	1.57 (1.12–2.54)	1.13–3.00
- MONOCYTES [10^3^/µL]	0.53 (0.36–0.68)	0.22–0.63
- EOSINOPHILS [10^3^/µL]	0.09 (0.04–0.18)	0.03–0.29
- BASOPHILS [10^3^/µL]	0.03 (0.02–0.05)	0.02–0.07
- IG [10^3^/µL]	0.02 (0.01- 0.04)	0.01–0.04
- PLT [10^3^/µL]	243.0 (188.0–287.0)	153–368
- RE-LYMP [10^9^/L]	0.10 (0.06–0.18)	0–0.5 × 10^9^/L
- **RE-LYMP** [%]	**6.5 (4.10–11.70) ↑**	**0–5%**

Abbreviations: f, female; m, male; IG, immature granulocyte count; PLT, platelets; RE-LYMP, reactive lymphocytes, **↑** above the reference value.

**Table 2:** Proportion of Sysmex morphological parameters in patients: with positive COVID-19 test—group A, control group (HC)—group B and the group with other virus infections with negative COVID-19 test—group C. Data expressed as median (Q1–Q3).

Sysmex Parameters [Median (Q1–Q3)]	A. COVID-19(+) *n* = 20	B. HC *n* = 20	C. COVID-19(−) Viruses *n* = 20	* *p* < 0.05 A-B-C Anova Kruskal-Wallis	* *p* < 0.05 in Groups Post-Hoc
- WBC [10^3^/µL]	4.25 (3.59–6.72)	6.08 (5.06–8.02)	6.72 (4.14–9.34)		
- NEUTROPHILS [10^3^/µL]	2.84 (2.20–3.38)	3.44 (3.12–4,70)	3.51 (2.18–4.90)		
- LYMPHOCYTES [10^3^/µL]	0.85 (0.69–1.56)	1.72 (1.52–2.32)	1.95 (1.25–2.93)	* *p* = 0.0250	A-B, A-C
- MONOCYTES [10^3^/µL]	0.36 (0.26–0.64)	0.56 (0.42–0.69)	0.57 (0.36–0.69)		
- EOSINOPHILS [10^3^/µL]	0.05 (0.00–0.08)	0.18 (0.07–0.26)	0.10 (0.05–0.16)	* *p* = 0.0038	A-B
- BASOPHILS [10^3^/µL]	0.02 (0.02–0.04)	0.04 (0.02–0.05)	0.04 (0.02–0.06)	* *p* = 0.0003	A-B
- IG [10^3^/µL]	0.02 (0.01–0.09)	0.01 (0.01–0.02)	0.02 (0.01–0.04)		
- PLT [10^3^/µL]	196.00 (177–290)	261.50 (230–287)	226.50 (190–286)		
- RE-LYMP [10^9^/L]	0.05 (0.04–0.09)	0.08 (0.05–0.10)	0.21 (0.13–0.37)		
- RE-LYMP [%]	5.45 (2.80–8.20)	4.20 (3.10–5.00)	11.05 (7.75–25.2)	* <0.0001	A-C, B-C

Abbreviations: HC, healthy control; IG, Immature Granulocyte count; PLT, platelets; RE-LYMP, reactive lymphocytes; WBC, white blood cell count, * p < 0.05 statistically significant.

**Table 3:** Differences of basic lymphocyte subtypes and plasmablasts between patients: with positive COVID-19 test—group A, control group (HC)—group B and group with other virus infections with negative COVID-19 test—group C. Data expressed as median (Q1–Q3).

Lymphocyte Subset (%) [Median (Q1–Q3)]	A. COVID-19(+) *n* = 20	B. HC *n* = 20	C. COVID-19(−) Viruses *n* = 20	* *p* < 0.05 A-B-C Anova	* *p* < 0.05 in Group Post Hoc
Lymphocytes subsets: (% of all cells)					
Lymphocytes [%] [10^3^/µL]	32.6 (21.1–49.3) 0.98 (0.76–2.99)	39.7 (34.2–44.7) 2.16 (1.75–2.73)	36.9 (28.7–45.5) 2.25 (1.64–3.08)	* 0.0350	A-B, A-C
Lymphocytes T [%] [10^3^/µL]	24.3 (13.9–37.5) 0.65 (0.57–2.24)	29.6 (25.6–35.0) 1.73 (1.39–2.13)	28.1 (23.6–34.3) 1.88 (1.25–2.40)	- -	
Lymphocytes T CD4 [%] [10^3^/µL]	13.3 (6.3–23.1) 0.48 (0.26–1.11)	18.8 (16.1–20.7) 1.04 (0.84–1.27)	16.5 (12.8–26.0) 1.08 (0.70–1.38)	- -	
Lymphocytes T CD8 [%] [10^3^/µL]	9.9 (4.2–12.6) 0.33 (0.16–0.86)	11.7 (8.1–14.4) 0.70 (0.50–0.90)	13.5 (10.1–7.4) 0.57 (0.45–0.85)	- -	
CD4/CD8	1.3 (1.0–3.5)	1.4 (1.3–2.1)	1.5 (1.3–0.7)	-	
Lymphocytes B [%] [10^3^/µL]	2.0 (1.4–4.7) 0.13 (0.03–0.18)	3.3 (2.5–4.1) 0.23 (0.12–0.28)	2.8 (2.0–4.2) 0.20 (0.12–0.28)	- -	
NK cells [%] [10^3^/µL]	5.0 (4.1–9.1) 0.18 (0.10–0.40)	3.4 (2.5–5.6) 0.21 (0.13–0.38)	5.0 (2.5–6.5) 0.25 (0.14–0.44)	- -	
**Plasmablasts** (% of B CD19+ cells)	8.8 (6.1–26.5)	2.7 (1.8–3.5)	11.1 (2.2–26.2)	* 0.0001	* A-B * B-C

Abbreviations: HC healthy control; RE-LYMP, reactive lymphocytes, * p < 0.05 statistically significant.

**Table 4:** Differences of CD4+ and CD8+ lymphocytes T with expression of CD25+, CD45RO+, HLA-DR+ or CD38+ between patients: with positive COVID-19 test—group A, control group (HC)—group B and group with other virus infections with negative COVID-19 test—group C. Data expressed as median of percentage or geometric mean fluorescence (GMF) intensity (Q1–Q3).

Lymphocyte Subset (%) [Median (Q1–Q3)]	A. COVID-1(+) *n* = 20	B. HC *n* = 20	C. COVID-19(−) Viruses *n* = 20	* *p* < 0.05 A-B-C Anova	* *p* < 0.05 in Group Post Hoc
**CD4+ subpopulation:** (% of CD4+ cells)					
CD4+ CD25 %	3.0 (2.0–6.5)	19.8 (13.2–29.1)	24.1 (20.3–33.2)	* 0.0000	* A-C * A-B
CD4+ CD25 GMF	108.0 (96.0–135.0)	304.5 (229.5–352.0)	290.0 (251.0–366.5)	* 0.0120	* A-C * A-B
CD4+ CD45RO %	65.7 (52.2–75.3)	73.2 (61.3–82.3)	73.3 (61.4–77.0)	-	-
CD4+ CD45RO GMF	2437.5 (1647.0–4373.0)	4807.5 (3194.5–7098.5)	5280.5 (3651.0–7793.5)	* 0.0129	* A-C * A-B
CD4+ HLA-DR+ %	6.1(4.2–12.9)	10.7 (8.3–14.5)	8.7 (7.0–31.0)	-	-
CD4+ HLA-DR+ GMF	118.0 (105.0–158.0)	137.5 (124.5–159.0)	148.5 (122.5–386.0)	-	-
CD4+ CD38+ %	11.0 (9.0–13.5)	18.0 (11.9–27.4)	23.0 (16.5–53.1)	* 0.0050	* A-C
CD4+ CD38+ GMF	313.0 (262.0–390.0)	232.5 (185.5–286.0)	317.0 (216.5–865.0)	-	-
**CD8+ subpopulation:** (% of CD8+ cells)					
CD8+ CD25+ %	0.2 (0.0–1.2)	2.5 (0.9–4.5)	3.4 (1.4–6.6)	* 0.0009	* A-C * A-B
CD8+ CD25+ GMF	80.5 (63.0–101.0)	121.0 (112.0–138.0)	127.5 (119.0–144.5)	* 0.0001	* A-C * A-B
CD8+ CD45RO+ %	30.0 (20.8–35.9)	65.0 (55.9–71.5)	51.9 (42.9–66.4)	* 0.0001	* A-C * A-B
CD8+ CD45RO+ GMF	796.0 (571.0–1293.0)	2683.5 (1922.0–3735.5)	2136.0 (1313.5–3061)	* 0.0002	* A-C * A-B
CD8+ HLA-DR+ %	22.5 (14.5–31.3)	31.5 (23.3–34.3)	28.2 (18.6–76.3)	-	-
CD8+ HLA-DR+ GMF	200.5 (165.0–307.0)	262.5 (206.5–298.0)	246.0 (183.0–3613.5)	-	-
CD8+ CD38+ %	23.3 (15.1–49.5)	6.3 (4.3–11.0)	19.6 (5.4–80.0)	* 0.0222	* A-B
CD8+ CD38+ GMF	349.0 (233.0–589.0)	137.0 (127.0–177.0)	229.0 (131.5–2000.5)	* 0.0050	* A-B

Abbreviations: GMF, geometric mean fluorescence; HC, healthy control; RE-LYMP, reactive lymphocytes, * p < 0.05 statistically significant.

## Appendix A

**Table A1:** COVID-19 patients’ characteristics.

ID	Age	f/m	Symptoms	PEMC	Oxygen Suplementation
1	80	m	Fever, dyspnea, diarrhea, fatigue	yes	no
2	78	f	Fever, cough, dyspnea, fatigue	yes	yes
3	63	m	Fever	yes	yes
4	42	m	Fatigue	no	no
5	39	f	Fatigue	no	no
6	44	m	Fever, cough, dyspnea,	yes	no
7	37	f	Fever, cough, dyspnea, diarrhea	yes	no
8	35	f	Fever, cough, dyspnea, fatigue	no	no
9	57	m	Fever, cough, dyspnea,	no	no
10	78	f	Fever, cough	yes	no
11	39	f	Fatigue	yes	no
12	72	m	Fever, cough, dyspnea, diarrhea, fatigue	yes	yes
13	28	m	Fever, cough, dyspnea, fatigue	no	no
14	63	m	Fever, cough	yes	no
15	43	m	Fatigue	yes	no
16	33	m	Fever, cough, diarrhea, fatigue	no	no
17	67	f	Fever, cough, dyspnea, fatigue	no	yes
18	72	m	Fever, cough, dyspnea, diarrhea, fatigue	no	no
19	47	f	Fever, cough, dyspnea, diarrhea, fatigue	no	no
20	34	m	Fever, cough, dyspnea, diarrhea, fatigue	no	no

Abbreviations: f: female; m: male; PEMC: pre-existing medical conditions.
